# Bridging the gap between tradition and innovation in psychotherapy: The promise of awareness integration theory

**DOI:** 10.1371/journal.pmen.0000095

**Published:** 2024-08-13

**Authors:** Foojan Zeine

**Affiliations:** 1 Awareness Integration Institute, San Clemente, California, United States of America; 2 Department of Health Science, California State University at Long Beach, Long Beach, California, United States of America; PLOS: Public Library of Science, UNITED KINGDOM OF GREAT BRITAIN AND NORTHERN IRELAND

In an era marked by unprecedented challenges to mental health and wellbeing, the demand for effective therapeutic interventions has never been greater. In 2019, a global estimate of 970 million individuals were affected by mental health conditions, with anxiety and depression being the most prevalent [1]. The rate of depression diagnoses among U.S. adults has increased considerably, with 29.0% indicating they have been diagnosed with depression at some stage in their lives, marking an almost 10-percentage point rise. Additionally, the proportion of Americans currently experiencing, or receiving treatment for, depression has escalated to 17.8% since 2015[2]. In the first year of the COVID-19 pandemic, the global prevalence of anxiety and depression surged by 25%, as reported in a scientific brief by the World Health Organization (WHO). Despite 90% of surveyed countries incorporating mental health and psychosocial support into their COVID-19 response plans, significant gaps and concerns persist [1]. According to a research study of American teens (aged 12–15 years), those who used social media for more than three hours each day were two times more likely to experience adverse mental health outcomes, including depression and anxiety symptoms [3]. The support that has been available has not been effective.

The current mental health crisis highlights the need for innovative and comprehensive therapeutic approaches. Awareness Integration Theory (AIT) emerges as a promising framework for fostering transformative change through its wide-ranging approach to integrating cognitive, emotional, behavioral, and somatic interventions. This theory is adaptable to traditional in-person psychotherapy, telehealth, educational systems, and mobile app practices. AIT represents a comprehensive psychotherapeutic and psychoeducational model designed to enhance self-awareness and emotional regulation, remove psychological barriers, and facilitate tangible improvements across various life domains. Grounded in psychology and neuroscience, AIT draws upon diverse theoretical frameworks, including Cognitive Behavioral Therapy (CBT), Humanistic/Existential Theories, Emotion-Focused Therapy (EFT), Psychodynamic, Solution-Focused Therapy, trauma-informed, and mindfulness practices. By integrating these approaches into a unified, open-structured framework, AIT provides a streamlined pathway, which aims to facilitate living a fulfilled and healthy life [4].

Through a six-phase intervention process, AIT guides individuals on a journey of self-awareness, discovery, and empowerment, facilitating transformative change at a deep and lasting level.

**Phase 1**: This phase focuses on raising awareness of the participant’s cognitive processes, emotional responses, and behavioral patterns and how these influence daily experiences. Questions probe perceptions and attitudes toward others, revealing generalized belief systems and internal dualities.

**Phase 2**: The second phase enhances awareness of subjective projections about others’ opinions and perceptions. It strengthens understanding of internal processes and assesses the impact of these projections on the participant’s life.

**Phase 3**: This phase fosters a reflective understanding of identity, exploring core beliefs about one self. Participants answer questions about self-perception, emotions, and self-treatment, and the impact on their life.

**Phase 4**: This phase identifies negative and irrational core beliefs that create limitations in the client’s life and dismantles them into neutral/positive and functional beliefs and attitudes in every area of life. The participant identifies the initial formation of these negative beliefs, connects their present self with their wounded past, and integrates power and resilience into a cohesive whole. AIT promotes a sense of accountability and responsibility regarding one’s attitude and life outcomes. This sense of accountability fosters empowerment and fulfillment and reduces the victimhood stance.

**Phase 5**: This proactive phase guides the participant through visualization exercises to cultivate a new self-concept. Emphasis is on nurturing a positive attitude and sense of agency. Participants explore values and beliefs, contemplating their desired identity, commitments, goals, and aspirations. AIT integrates healthy values, thoughts, intentions, feelings, and behaviors, to envision the future and intentional action in the present time in all domains of life.

**Phase 6**: The final phase involves structuring a daily functional action plan and support system to sustain healthy thought, behavioral patterns, and emotional regulation toward living a fulfilled life [4].

Empirical studies have demonstrated the effectiveness of AIT in reducing symptoms of depression, anxiety, and PTSD while simultaneously enhancing self-esteem, self-efficacy, and overall wellbeing. The Personal Growth Institute explored the effects of partaking in AIT-based face-to-face therapy and reported a decrease in depression by 76% and anxiety by 60%. Simultaneously, they reported an increase self-esteem by 43% and self-efficacy by 20% [5]. Furthermore, AIT was offered to separated or divorced individuals who took a pre and posttests in a 6-hr. workshop setting with a promising result of 27.5% improvement in depressive moods and a 37% decrease in feelings of anxiousness and anxiety. In addition, there was a 15% increase in self-esteem, and a 13% boost in self-efficacy [6]. Telehealth has emerged as a vital tool for delivering mental health care, particularly considering the COVID-19 pandemic disruptions to traditional care delivery models. And indeed, studies utilizing AIT via telehealth reported a decrease in anxiety by 50% and an increase in self-esteem by 60% [7]. In another telehealth case study, depression and anxiety scoring decreased by 66%, and 75%, respectively whilst post-traumatic stress disorder (PTSD) symptoms were reduced by 66% [8].

The global landscape of mental health care is undergoing a paradigm shift, with increasing emphasis on innovative approaches that transcend traditional therapeutic modalities. Mobile mental health apps offer unique potential, such as providing timely, cost-effective support, bypassing the stigma surrounding help-seeking, and enhancing treatment outcomes [9]. A study on the American college student population using the Awareness Integration Theory as a self-help model showed an overall 68% decrease in depression and a 21.72% decrease in anxiety [10]. Amidst this evolution, the Foojan app emerged as an innovative telehealth platform that leverages the principles of AIT to deliver personalized, affordable, accessible, and practical mental health support worldwide, offering a holistic framework that integrates diverse psychological interventions to catalyze transformative change. Through resources such as the Foojan app, users can access efficient, affordable daily practice of guided self-awareness journaling, access skill-building videos, and connect with professionals for individual psychotherapy or coaching sessions. Foojan app user data has shown improvements in diverse domains of life as shown on Table 1 [11].

**Table 1:** Data from “foojan” app from 280 users during 2/2023-7/2024. www.foojan.com.

Life Area	Pre-survey	Post-survey	% of Improvement
Self	26%	93%	67%
Intimate Relationship	22%	88%	66%
Mother	25%	86%	61%
Father	20%	84%	64%
Finance	28%	85%	57%
Career	25%	90%	65%
Sister	22%	84%	62%
Brother	25%	82%	57%
Mother-in-law	20%	82%	62%

Sample Data

These numbers are averages collected from 280 users during Feb 2023 until Jul. 2024.

According to the Centers for Disease Control and Prevention, mental health literacy, mindfulness, social, emotional, and behavioral learning, and psychosocial skill building are proven in-school strategies to promote and support mental health and wellbeing [12]. AIT has been adapted to be utilized as a proactive psycho-emotional-social-educational and skill-building model from preschools to colleges in the United States and Europe [13].

In conclusion, the incidence of mental health issues is increasing, highlighting an urgent requirement for enhanced prevention and treatment methods [14]. AIT represents a transformative framework for fostering mental health and wellbeing through its holistic approach to integrating diverse psychological, emotional, and behavioral interventions. This model is adaptable in educational institutions (from preschool to universities) as a prevention and healthy lifestyle strategy, self-help improvement and skill-building via the Foojan app, and psychotherapy (in-person and telehealth) as a treatment model. By bridging the gap between traditional and innovative therapeutic modalities, AIT offers a comprehensive and personalized approach to mental health care, addressing the growing demand for efficient, effective, and affordable interventions in today’s challenging landscape.
